# Phytoplankton Chytridiomycosis: Fungal Parasites of Phytoplankton and Their Imprints on the Food Web Dynamics

**DOI:** 10.3389/fmicb.2012.00361

**Published:** 2012-10-12

**Authors:** Télesphore Sime-Ngando

**Affiliations:** ^1^UMR CNRS 6023, Laboratoire Microorganismes: Génome et Environnement, Clermont Université Blaise PascalClermont-Ferrand, France

**Keywords:** fungi, chytrids, microbial parasites, phytoplankton hosts, food webs, microbial ecology, aquatic ecosystems

## Abstract

Parasitism is one of the earlier and common ecological interactions in the nature, occurring in almost all environments. Microbial parasites typically are characterized by their small size, short generation time, and high rates of reproduction, with simple life cycle occurring generally within a single host. They are diverse and ubiquitous in aquatic ecosystems, comprising viruses, prokaryotes, and eukaryotes. Recently, environmental 18S rDNA surveys of microbial eukaryotes have unveiled major infecting agents in pelagic systems, consisting primarily of the fungal order of Chytridiales (chytrids). Chytrids are considered the earlier branch of the Eumycetes and produce motile, flagellated zoospores, characterized by a small size (2–6 μm), and a single, posterior flagellum. The existence of these dispersal propagules includes chytrids within the so-called group of zoosporic fungi, which are particularly adapted to the plankton lifestyle where they infect a wide variety of hosts, including fishes, eggs, zooplankton, algae, and other aquatic fungi but primarily freshwater phytoplankton. Related ecological implications are huge because chytrids can killed their hosts, release substrates for microbial processes, and provide nutrient-rich particles as zoospores and short fragments of filamentous inedible hosts for the grazer food chain. Furthermore, based on the observation that phytoplankton chytridiomycosis preferentially impacts the larger size species, blooms of such species (e.g., filamentous cyanobacteria) may not totally represent trophic bottlenecks. Besides, chytrid epidemics represent an important driving factor in phytoplankton seasonal successions. In this review, I summarize the knowledge on the diversity, community structure, quantitative importance, and functional roles of fungal chytrids, primarily those who are parasites of phytoplankton, and infer the ecological implications and potentials for the food web dynamics and properties. I reach the conclusion that phytoplankton chytridiomycosis represents an important but as yet overlooked ecological driving force in aquatic food web dynamics and network organization.

## Introduction

Parasitism is one of the earlier known and most common ecological interactions in nature (Cavalier-Smith, 1993), occurring in almost all environments (Lafferty et al., 2006). Parasites have received much more attention in terrestrial than in aquatic ecosystems (Kuris et al., 2008), where they represent a strong forcing factor for critical evolutionary and ecological processes, e.g., population dynamics, species successions, competition for resources, species diversification, and energy and gene flows (Hudson et al., 2006). Few attempts have been made to include parasites in the food web dynamics of aquatic systems (McCallum et al., 2004; Amundsen et al., 2009), with special emphasis on parasites of plants (Buschmann et al., 2001), invertebrates (Perkins, 1993), and vertebrates (Marcogliese, 2004).

Recent ecological and molecular surveys in pelagic environments have revealed a high occurrence of eukaryotic putative parasitoids, especially in the picoplanktonic size-fraction (López-García et al., 2001; Lefranc et al., 2005; Lefèvre et al., 2007, 2008), adding to the other typical parasitic entities such as viruses (Sime-Ngando and Colombet, 2009). Eukaryotic parasites known from 18S rDNA diversity surveys include fungal members of the Phylum Chytridiomycota as a major water-borne group, comprising both host-attached vegetative (i.e., sporangia) and free-swimming infective (i.e., flagellated zoospores) stages (Gleason and MacArthur, 2008). The Phylum Chytridiomycota (thereafter, chytrids) occupies the basal branch of the Kingdom Fungi and because associated members are small in size and lack conspicuous morphological features, chytrids are hardly distinguishable from many flagellated protists such as the sessile choanoflagellates or bicosoecids which are bacterivores (Lefèvre et al., 2007, 2008). Chytrids exhibit different trophic strategies (i.e., parasitism, saprotrophy) than these phagotrophic protists. In addition, chytrid propagules can represent key intermediates in the food chain (Gleason et al., 2009). Indeed, fungal zoospores have suitable dimensions and represent a valuable food source for zooplankton. Similar to some protists (Desvilettes and Bec, 2009), fungal zoospores also contain essential fatty acids that might further upgrade the nutritional quality of food ingested by zooplankton such as *Daphnia* (Kagami et al., 2007a,b).

It is thus important to include eukaryotic parasites in the microbial ecology of aquatic environments. This is timely and will help to integrate “novel” ecological perspectives and extend the concept of parasitism and their functional potential to the aquatic food web dynamics (Gachon et al., 2010; Sime-Ngando and Niquil, 2011). This review focuses primarily on chytrid parasites of phytoplankton, and complements our recent review on eukaryotic microbial parasites in the plankton (Rasconi et al., 2011), by summarizing the knowledge on their diversity, community structure, quantitative importance, and ecological roles, and infer the ecological implications and potentials for the food web dynamics, properties, and overall topology.

## Chytrids: A Disregarded Diversity within the Kingdom of Eumycota

**Figure 1:**
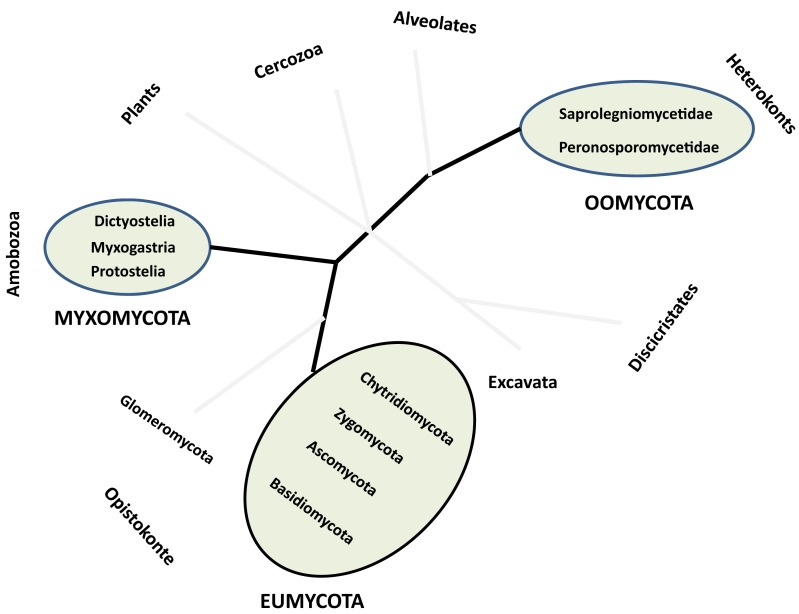
**Approximate position of true-Fungi (Eumycota) and fungus-like (Oomycota and Myxomycota) organisms in a schematic phylogenetic tree of eukaryotes**. Modified from Jobard et al. (2010a).

The term Fungi globally embraces all organisms that belong to the kingdom Eumycota (i.e., the so-called true-fungi), while the term fungi also includes other microorganisms (i.e., fungus-like organisms) traditionally studied by mycologists such as members of Myxomycota (Amobozoa) also called slime molds, and of Oomycota (Heterokonts) also called water molds, as well as mushrooms and other molds (Figure 1). All these organisms share similar trophic strategies, namely saprophytism, parasitism, and other symbiotic associations, and can occur in the same ecosystem (reviewed in Jobard et al., 2010a; Wurzbacher et al., 2010, 2011). They constitute one of the last frontiers of the undiscovered biodiversity and the related functions that challenge aquatic microbial ecology today. The number of fungi present on earth was estimated to about 1.5 million species, from which approximately 97,000 have so far been identified, including about 300 species of anamorphic fungi and 300 species of Ascomycota but only few species of Basidiomycota and Chytridiomycota. Known fungal species correspond mostly to cultured specimens thriving in moist soils, lotic systems, mangroves, and wetlands, or to economically interesting pathogens of humans, plants, and animals. For example, *Batrachochytrium dendrobatidis*, the chytridiomycosis agent of one of the most deadly contemporary skin diseases that drive the decline of amphibian populations worldwide has been well studied (Voyles et al., 2009), leading to a great deal of publicity.

The kingdom Eumycota groups the following four divisions: Ascomycota, Basidiomycota, Zygomycota, and Chytridiomycota (James et al., 2006), but also include a particular group of microorganisms phylogenetically affiliated to the divisions Ascomycota and Basidiomycota (i.e., the so-called Deuteromycota), known as “fungi imperfecti” because only asexual forms (or anamorphs) have been observed during their life cycle. This group contains the particularly well studied members of Fungi in running waters, i.e., the hyphomycetes (Shearer et al., 2007). Zygomycota and Chytridiomycota do not emerge as monophyletic groups in recent phylogenetic analyses (James et al., 2000, 2006). Taxa traditionally placed in Zygomycota are distributed among Glomeromycota (Figure 1) and several subphyla *incertae sedis*, including Mucoromycotina, Entomophtoromycotina, Kickxellomycotina, and Zoopagomycotina. The Chytridiomycota is retained in a restricted sense, with Neocallimastigomycota and Blastocladiomycota representing segregate phyla of flagellated Fungi, also known as zoosporic Fungi. These zoosporic true-fungi (chytrids) were described according to modes of reproduction, thallus development and ecology and, most importantly, morphological characteristics of the thallus such as size, shape, and ornamentation of the sporangia, presence or absence of an operculum, and rhizoidal arrangement (Blackwell et al., 2006). Because they rely on free water phase for whole or part of their life cycle, chytrids are considered typical pelagic (i.e., floating) species.

## Ecological Conceptualization of Chytrid Life Cycle and Adaptation to Pelagic Lifestyle

**Figure 2:**
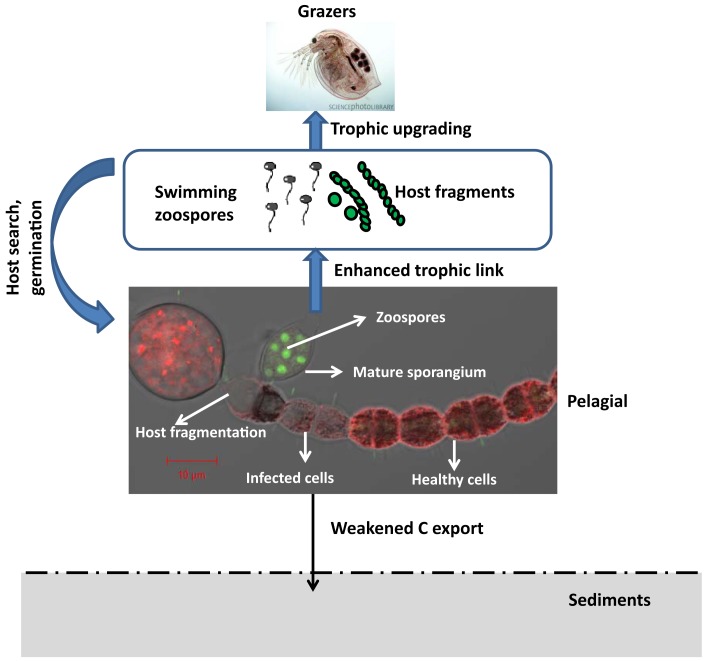
**Phytoplankton chytridiomycosis affects food web dynamics and properties by killing part of or the entire host filaments or free-living cells, and provide nutrient-rich particles as zoospores and short fragments of filamentous inedible hosts for the grazer (*Daphnia* in this case) food chain**. Because chytrids preferentially attack larger size species such as the filamentous cyanobacterium *Anaebaena macroscopora* in this case, blooms of such species may not totally represent trophic bottleneck to be sequestered in the sediments. Part of their energy are retained in the pelagial and recycled through the trophic cascade. Source for Daphnia zooplankton micrograph is the science photo library: http://www.sciencephoto.com/media/367262/enlarge. The microphotograph of infected *A. macroscopra* filament with Cytox Green stained zoospores observed under confocal microscopy is a courtesy of Melanie Gerphagnon.

Chytrid species have an interesting life cycle in the context of the pelagic realm where the two main stages (i.e., sporangium and zoospore) have different effects on the food web dynamics. Most members reproduce asexually by releasing zoospores with a single posteriorly directed whiplash flagellum (Sparrow, 1960; Barr, 2001). In a few species of the Neocallimastigales, zoospores are multiflagellate (Trinci et al., 1994) or in at least one species of the Blastocladiales (Hoffman et al., 2008) and one species in the Monoblepharidales (Ustinova et al., 2000), the spores lack flagella. The thallus can be either monocentric, polycentric, or filamentous (hyphal; Sparrow, 1960) and are able to grow either on top or within substrates. In the typical life cycle, a free-living zoospore encysts to the host and expands intracellularly as a tubular rhizoid, i.e., the nutrient conveying system for the formation of fruit bodies (i.e., the infective sporangium) from which propagules (i.e., motile zoospores) are released into the environment. The hosts of parasitic chytrids in aquatic systems are highly diverse, including both prokaryotic (i.e., cyanobacteria) and eukaryotic phytoplankton, protists, invertebrates (larvae of insects, rotifers, nematodes, crustacean such as copepods, ostracods, cladocera etc…), flowering plants or other fungi. Chytridiomycosis epidemics are known to produce massive amount of zoospores, now known as valuable food source for zooplankton (Kagami et al., 2007a,b). The two main development stages of chytrids thus highlight two overlooked ecological potentials in the food web dynamics: (i) parasitic predation of host populations, most of which are inedible (i.e., unexploited by grazers), and (ii) the subsequent trophic link via the release of suitable zoospore food for zooplankton. In addition, we have recently shown that chytrid parasitism of cells within the filaments of cyanobacteria during bloom events can result in a mechanical fragmentation of the inedible filaments into shorter-size edible filaments (Gerphagnon et al., submitted), thereby enhanced the contribution of fungal parasites to the bloom decline (Figure 2).

Chytrids are more commonly found growing in bodies of waters, primarily in freshwater environments, and in soils as well. In general, chytrids prefer environments with low osmotic potentials. Only three species (i.e., *Rhizophydium littoreum*, *Thalassochytrium gracilariopsis*, and *Chytridium polysiphoniae*) have been properly identified and partially characterized from brackish and marine ecosystems. These species are either facultative or obligate parasites of marine macroalgae and invertebrates. Also, some species of *Olpidium* and *Rhizophydium* are parasites of small marine green algae and diatoms (review in Gleason et al., 2011). Water-borne fungi have to face various difficulties and constraints characteristic of aquatic habitats, among which oxygen availability perhaps may be one of the most restrictive parameters. Except for Neocallimastigales in which all species are amitochondrial obligate anaerobes known from the rumen hindguts of herbivorous mammals where ambient temperatures approach 40°C, all chytrids are obligate aerobes and their growth rates are greatly inhibited by low dissolved oxygen concentrations (Gleason et al., 2008). Investigations of the tolerance to anoxia in the chytrids *Rhizophydium sphaerotheca* and *Phlyctochytrium punctatum* have revealed that these fungi are facultative anaerobes (Goldstein, 1960).

Adaptation to dispersal in running water is typical of some fungi which have developed spores with particular morphologies (e.g., large and multiradiate conidia of hyphomycetes) allowing successful attachment on substrates in flowing waters (Jobard et al., 2010a). Chytrids represent the sole true-fungi phylum with species producing flagellated cells in their life cycle. These temporary swimming life zoospores are particularly well adapted to dispersal in pelagic medium where chytrids represent the best studied group of fungi, primarily in lakes where they occur mainly as phytoplankton parasites (Ibelings et al., 2004; Rasconi et al., 2009, 2012). However, these studies remain limited, mainly because of methodological constraints and under sampling of pelagic ecosystems, where the occurrence of fungi is often considered as contamination from allochthonous watershed inputs (Goh and Hyde, 1996; Jobard et al., 2010a).

## Methodological Limitations

Conventional methods for analysis of zoosporic fungi to date have mainly relied on direct observation and baiting techniques, with subsequent fungal identification using morphological characteristics (review in Marano et al., 2012). Earlier descriptions of chytrid parasites were based on microscopic observation of sporangia which exhibit morphological features that can allow approximate phenotypic identification of specimens in living samples or those preserved with Lugol’s iodine (Rasconi et al., 2011). Such approaches have provided detailed descriptions of the morphological features using light or phase-contrast microscopy (Ingold, 1940; Canter, 1949, 1950, 1951). Subsequently, electron microscopy was used to describe different life stages, and a number of studies describing the ultrastructural cytology of fungal zoospores and spore differentiation are available (Rasconi et al., 2011). The chytrid *Blastocladiella* sp. was the first fungal model for detailed structural studies on sporogenesis (Lovett, 1963). The precise conformation of the flagellar rootlets and the spatial distribution of organelles in zoospores have been determined, providing the basis for chytrid taxonomy (review in Gleason and Lilje, 2009).

Ecological investigations of the dynamics of chytrid populations in natural environments have been improved more recently with epifluorescence microscopy. Several fluorochromes have been used, among which the chitin stain calcofluor white (CFW) penetrates infected host cells remarkably well and is more efficient for the observation and photomicrography of the complete rhizoidal system of parasites, which is an important criterion for chytrid identification (Rasconi et al., 2009; Sime-Ngando et al., 2012a).

Molecular biology techniques have allowed a thorough reconstruction of chytrid phylogenies (James et al., 2006) and are increasingly providing more specific tools, primarily oligonucleotide probes, for the quantitative ecological study of aquatic parasites. Fluorescent *in situ* hybridization, amplified with horseradish peroxidase activation by fluorescent tyramide (also known as catalyzed reporter deposition, CARD FISH or TSA FISH), is a reliable approach to detect and count specific chytrid parasites, especially the zoospore stages that lack a chitinaceous wall, precluding any simple use of fluorochromes such as CFW (Sime-Ngando et al., 2012b). The clone-FISH approach, which was originally designed for prokaryotes, was recently adapted by Jobard et al. (2010b) to the assessment of zoosporic fungi in natural samples. Clone-FISH is based on the genetic modification of a clone of *Escherichia coli* by inserting plasmid vector containing the target 18S rDNA sequence. The main advantage of the clone-FISH method is that pure cultures which are necessary to validate the specificity and hybridization conditions of FISH or CARD FISH probing are not needed (Jobard et al., 2010b). Oligonucleotidic probes have also been used in quantitative real-time PCR (qPCR), which is an excellent tool for solving the limitations in the detection of less abundant and rare species (Lefèvre et al., 2010; Sime-Ngando and Jobard, 2012). Further application of PCR-base methods, primarily of next generation sequencing technologies, will not only advance our quantitative understanding of zoosporic fungal ecology and diversity, but also their function through the analysis of their genomes and gene expression (Monchy et al., 2011). Nevertheless, it is still necessary to complement these molecular-based approaches with cultivation-based methods in order to gain a fuller understanding of the ecological and physiological roles of zoosporic fungi (Marano et al., 2012).

The methodological difficulties are thus increasingly being overcome, and it is becoming evident that techniques from molecular biology are useful for the study of zoosporic fungi. The challenge of matching molecular sequences to microscopic phenotypes is also on the route to be tackled (Jobard et al., 2012; Monchy et al., 2012). Furthermore, to assess the functional impact of chytrid parasites on host populations, algorithms commonly used by parasitologists such as the prevalence and the intensity of infection have been applied in ecological studies of chytridiomycosis (Rasconi et al., 2012). These parameters are derived from direct microscopy and are critical for assessing the community structure of parasites, interactions with hosts, and epidemiology, as well as the potential impact of fungal parasites on the food web dynamics. Understanding the environmental factors that induce epidemics can also be inferred this way (i.e., through empirical correlations) or by using epidemiological approaches such as the changes in incidence rates (i.e., the number of new cases of infections occurring during a given time) or the occurrence of epidemics (i.e., a widespread outbreak of an infection) within host populations (Fox, 2003).

## Seasonal Diversity, Abundance, Community Structure and Trophodynamics of Chytrids

Studies on pelagic chytrids started in the British lakes (Cook, 1932), and different authors have provided descriptions of morphological characters (Reynolds, 1940; Canter and Lund, 1948, 1953; Pongratz, 1966; Canter, 1972). Quantitative assessment of the importance of parasitism indicated that infection of diatoms, desmids, and other green algae is fairly common in freshwaters (Canter and Lund, 1948, 1968). However, most studies on parasite dynamics in natural plankton assemblages are focused on hosts or limited to the investigations of one or two fungal populations (Table 1). This makes the generalization of observed patterns difficult, and the seasonal dynamics of chytrids at the community level remain largely unknown.

**Table 1:** **Quantitative data on fungal parasites and parasitism of phytoplankton in temperate lake ecosystems**.

Environment, country (trophy)	Method	Sampling period (depth, m)	Chytrid sporangia (10^6^ l^−1^)	Sporangium biovolume (μm^3^)	Prevalence of infection (% infected host cells)	Intensity of infection (sporangia host cell^−1^)	Host (Chytrid)	Reference
Lake Pavin, France (O-M)	CFW staining and EM	Feb.–Dec. 2007 (Ze)	0.005–3.7	6.7–67.4	1.5–59.1	1–2.5	Phytoplankton communities (mixed)	Rasconi et al. (2012)
Lake Aydat, France (E)	CFW staining and EM	Feb.–Dec. 2007 (Ze)	0–3.4	8.7–72.4	0–98*	1–2	Phytoplankton communities (mixed)	Rasconi et al. (2012)
Lake Schöhsee, Germany (M)	Lugol staining and LM (Utermöhl)	Mar. 1987–May, 1989 (0–10)	ND	ND	0–>90** (≤1–10% of total host volume)	ND	Different species within Phytoplankton communities (mixed)	Holfeld (1998)
Lake Maarsseveen, The Netherlands (O-M)		1978–2010 (0–10)	ND	ND	0–90	ND	*Asterionella formosa (Rhizophydium planktonicum)*	Ibelings et al. (2004, 2011)
Lake Kinneret, Israel (O-M)	LM (Utermöhl)	Oct. 2000–Dec. 2003 (2 and 7)	ND	ND	0–83	ND	*Peridinium gatunense* (*Phlyctochytrium* sp.)	Alster and Zohary (2007)
Lake Schöhsee, Germany (M)	Lugol staining and LM (Utermöhl)	27 Jan.–7 Feb. 1989 (0–10)	ND	ND	∼10–80	∼0.1–1.8	*Stephanodiscus alpinus* (*Zygorhizidium* spp.)	Holfeld (2000)
Lake Suwa, Japan (E)	Dialysis tube cultures and LM	Nov. 1986–Nov. 1987 (0–4)	ND	ND	2–30	ND	*Asterionella formosa (Rhizophydium planktonicum or Zygorhizidium affluens)*	Kudoh and Takahashi (1990)
Shearwater, UK (E)		Several occasions between 1978 and 1981 (net samples)	ND	ND	0.2–1.4	Up to 4	Centric diatoms: *Cyclotella* spp., *Stephanodiscus hantzschii*, and *Melosira* spp. (mixed)	Sen (1988a)
Shearwater, UK (E)		10 epidemic periods between 1978 and 1980 (net samples)	ND	ND	15–90%	ND	*Microcystis aeruginosa* (*Rhizidium microcystidis*)	Sen (1988b)
Shearwater, UK (E)		Several occasions between 1978 and 1981 (net samples)	ND	ND	Up to 85%	ND	Several species of chlorophytes (mixed)	Sen (1988c)

*Recorded during a monospecific bloom of Anabaena flosaquae infected by Rhizosiphon crassum; **corresponds to the infection of Synedra acus infected by Zygorhizidium planktonicum.

*O, oligotrophic; M, mesotrophic; E, eutrophic or productive with recurrent cyanobacterial blooms; Ze, euphotic zone; CFW, calcofluor white (cf. Rasconi et al., 2009), EM, epifluorescence microscopy; LM, light microscopy; ND, not determined or not given explicitly*.

A recent extensive seasonal study in temperate freshwater lakes (Rasconi et al., 2012), based on CFW staining of infective sporangia and phenotypic identification, has identified up to 15 different chytrid species on diverse host populations, with specific biovolume ranging from 7 to 72 μm^3^ sporangium^−1^ (Table 1). Seasonal abundances increased from 0.0005 to 0.4 × 10^6^ sporangia l^−1^ in oligotrophic conditions to 0.0 to 32 × 10^6^ sporangia l^−1^ in eutrophic conditions (Table 1). In both conditions, sporangium abundances peaked with the development of preferential diatom hosts in spring and cyanobacteria in autumn, the autumn peak being largely higher in eutro- than in oligotrophic conditions, when a monospecific bloom of *Anaebaena* sp. occurred. Quantitative data on the seasonal abundance of zoospores are lacking.

All the 15 species identified were monocentric (i.e., with one center of growth and development) and eucarpic (using part of the thallus for the fruit-body formation and with a specialized rhizoidal system), typical of the class *Chytridiomycetes*, and belonged to two orders: the *Rhizophidiales* which contained one genus (*Rhizophidium*), and the *Chytridiales* which contained three genera (*Chytridium*, *Zygorhizidium*, and *Rhizosiphon*). The species of *Rhizophidium* spp. infected a wide diversity of hosts, including both large size (e.g., the Chlorophyta *Staurastrum* spp. and the diatoms *Asterionella formosa*, *Synedra* spp. and *Fragilaria crotonensis*) and small size algae (e.g., the diatom *Cyclotella* spp. and the Chlorophyta *Chodatella ciliata* and *Ankistrodesmus convolutus*). The species *Chitridium* spp. infected the chlorophyte *Oocystis* sp., the diatom *F. crotonensis*, and the cyanobacterium *Microcystis* sp., while the species of *Zygorhizidium* infected the diatoms *Melosira* spp. The genus *Rhizosiphon* comprised two species that are specific parasites of vegetative cells and akinetes (*R. crassum*) and of akinetes alone (*R. akinetum*), which correspond to different niches offer by the filamentous cyanobacterium host *Anabaena macrospora* in productive lakes (Gerphagnon et al., submitted). Although almost all the chytrid species were observed from oligo- to eutrophic conditions, the seasonal fungal community composition was largely dominated by species of the genus *Rhizophidium* (90% of total sporangium abundance) in oligotrophic conditions, and of the genera *Rhizophidium* (56%), *Zygorizidium* (22%), *Chytridium* (19%), and *Rhizosiphon* (14%) in eutrophic conditions (Rasconi et al., 2012).

The community structure of natural chytrids is intimately linked to the availability of hosts (Ibelings et al., 2004). However, except the study by Rasconi et al. (2012) that has proposed a general empirical model on chytrid seasonality and trophodynamics (i.e., with their hosts) based on the theoretical PEG model of seasonal succession of planktonic events in freshwaters (Sommer et al., 1986), there is still no study assessing the fungal species successions in natural environments. This contrasts with the general hypotheses and patterns of plankton (primarily phytoplankton and zooplankton) successions and community structure, which are well described in temperate lakes (Sommer et al., 1986). This was recently revisited by considering a suite of overlooked ecological interactions that included parasitism (Sommer et al., 2012). These authors concluded that the effects of these “novel” interactions on plankton seasonal succession are limited in terms of seasonal biomass patterns but strong in terms of species replacements.

In the Rasconi and coauthor’s model (Rasconi et al., 2012), during winter, the development and activities of both chytrid parasites and their phytoplanktonic hosts were at their lowest levels, because of low temperature, freezing, or ice-cover. From late winter on, the environmental conditions, primarily the increase in water temperature and in mixing-derived nutrient availability, favor the development of host communities, with the dominance of k-strategists (e.g., large diatoms) toward spring. As a consequence, the host – parasite contact probability increases, raising the chytrid infectivity and the production of large amount of zoospores. Enhanced infection prevalence then limits and provokes the decline of large diatoms, liberating niches for a diversified phytoplankton community of small size r-strategists. The abundance of chytrid sporangium reaches their lowest level, while the availability of food (i.e., small phytoplankton and fungal zoospores) favors the development of grazers and the establishment of a typical clear-water phase at the end of spring. During the summer months, favorable environmental conditions, together with a high grazing pressure, allow the development of a diversified and complex plankton community. Small edible hosts are inhibited by the grazing pressure, while the availability of large size hosts favors the proliferation of different chytrid species toward the end of the summer. From here, oligotrophic lakes significantly diverge from productive waters. In oligotrophic situations, autumnal overturn promotes species coexistence and phytoplankton diversity leads to the association of different species of chytrid parasites of chlorophytes and diatoms, but with general low infection prevalence due to a balanced host – parasite growth. In eutrophic lakes, nutrient conditions and persistent stratification favor the bloom of filamentous cyanobacteria from the end of the summer period toward early autumn, with the development of a monospecific community of chytrids (i.e., *Rhizosiphon* spp.). The highest infection prevalence is noted, followed by the decline of cyanobacteria – chytrid system toward the late seasonal phase (for more details, see figure number 7 in Rasconi et al., 2012).

## Phytoplankton Chytridiomycosis and the Influence of Environmental Factors

A basis for study chytrid epidemics within phytoplankton communities was recently provided for freshwater lakes where, in contrast to sporangium abundance and biomass that increased from oligo- to eutrophic conditions, the prevalence of infection is quite similar in both conditions, averaging about 20% (Rasconi et al., 2012). The highest prevalence (98%) was noted for the autumn bloom of a filamentous cyanobacterium (*A. macospora*) facing the parasite *Rhizosiphon crassum* in a productive lake. The host species composition and their size appeared as critical for chytrid infectivity, the larger hosts being more vulnerable, including pennate diatoms, desmids, and filamentous cyanobacteria. Such host species are apparently easier to hit because their size naturally increase the host – parasite contact rates, and are expected to excrete more attracting substances known to favor the zoospores searching of suitable hosts (Canter and Jaworski, 1981). Larger algae also contain more resources for the diet of parasites, and this is the common explanation of why algal species with larger cell size can be heavily infected, even at lower population density (Lund, 1957; Holfeld, 1998). On the other hand, the prevalence of infection was also shown to be correlated with total phosphorus, which may be related to the productivity of the milieu that offers good substrate conditions for the growth of parasite-host systems (Rasconi et al., 2012). The abundances but also the cell volumes of hosts thus seem important features in determining the amplitude of chytrid epidemics within natural phytoplankton. These parameters also appeared to be related with the tolerance threshold of infection, i.e., the critical prevalence or the level of prevalence from which the standing stock of phytoplankton starts to decline (Bruning et al., 1992). At low host abundance, the critical infection prevalence is generally lower that 20%, but increases with increasing host abundance. This is probably one of the mechanisms from which parasites regulate host populations.

Diverse environmental conditions, including temperature, turbulence, light, nutrient concentrations, and biotic factors such as predation but primarily the host availability, can influence the growth rate of fungal parasites (Canter and Jaworski, 1981; Bruning and Ringelberg, 1987; Bruning, 1991; Kagami et al., 2004). The primary factor determining the absence or the presence of a particular parasite in the environment is the availability of suitable hosts (Ibelings et al., 2004). The host population density is frequently considered an important factor in the ecology of parasites. Indeed, irrespective of temperature and light conditions, a minimal threshold value of the host density is required for the occurrence of epidemics (Bruning, 1991). Several studies have reported that parasites seem to grow better on healthy individuals within actively growing host populations (Canter and Lund, 1948; Van Donk and Ringelberg, 1983). However, this is far from being a generalization because evidence was also provided that epidemics in natural phytoplankton populations arise more easily when growth conditions for hosts are worst (Reynolds, 1984). Under such conditions, the growth rate of the algae would be relatively slow, contrasting with their fast-growing parasites. Rasconi et al. (2012) hypothesized that there are two different phases in the parasite – host trophodynamic: a synchronous growth phase of both chytrids and algae when high availability of hosts favored the encounter between parasites and newly produced sensible host cells, followed by a second phase corresponding to the decline of host populations due to the infection, characterized by an increase in the infection prevalence.

## Ecological Implications of Phytoplankton Chytridiomycosis

Based on the study by Rasconi et al. (2012), the prevalences of infection (% of infected host cells) typically average around 20%, with no significant variation with the trophic status of freshwater temperate lakes. These values increase to reach about 100% when monospecific blooms of infected hosts occurred in natural conditions or when specific chytrid-host systems are targeted in controlled conditions (Table 1). Chytrid infection commonly leads to the death of their host cells (Canter and Lund, 1951; Sen, 1988b,c; Kudoh and Takahashi, 1990; Bruning et al., 1992; Holfeld, 1998, 2000; Ibelings et al., 2004, 2011) and this is enhanced by the intensity of infection (number of parasites per host cell) which can largely exceed 1 (Table 1). Empty sporangia are currently found attached on dead phytoplankton cells (Rasconi et al., 2012), which is suggestive of the lethal issue of chytrid infection. There are several evidences that parasitism inhibits the development of sensible species, and particular attention has been paid to the occurrence of fungi on diatoms, and to the effects of parasitism on their seasonal distributions (Canter and Lund, 1948; Van Donk and Ringelberg, 1983). For example, in the oligotrophic Lake Pavin (France), the spring development of the diatoms *Asterionella* and *Synedra* was found to be inhibited by the chytrid *Rhizophidium planktonicum*. In productive Lake Aydat (France) another diatom, *Fragilaria*, became abundant but the proliferation of their parasites, *Rhizophidium* *fragilariae*, interrupted their development (Rasconi et al., 2012).

In natural phytoplankton community, the parasitized populations are often replaced by others with similar ecologically requirements, which can render unchanged the standing stocks of phytoplankton hosts in the ecosystem, with no visible obvious damage to the total community (Reynolds, 1940). However, chytrids seem to preferentially infect large and less edible phytoplankton species, as discussed previously.

Some examples provided in the literature suggest that large infected diatoms such as *Asterionella* sp. and *Fragilaria* sp. (mean length 50 and 70 mm, respectively) can be replaced by small centric diatoms such as *Stephanodiscus* spp. (10 mm; Van Donk and Ringelberg, 1983; Sommer, 1987). This implies that the development of large species is inhibited by infection, while smaller algae proliferate. Active parasitism may thus act on the host standing stock in a continuum from no change to significant changes but in all cases will affect the phytoplankton community structure. In the context of phytoplankton seasonal dynamics and species successions, this can have profound ecological implications (Van Donk, 1989). For example, due to chytrid parasites, the phytoplankton community can shift from a mature stage of development typically dominated by large, k-strategist species toward a pioneer stage of succession that favors the development of small, r-strategist species. In addition, by controlling the phytoplankton dynamics, chytrids can significantly affect the primary production of aquatic systems, as suggested by a negative correlation between the primary production and the per sporangium biovolume of chytrids in lakes (Rasconi et al., 2012).

More importantly, phytoplankton chytridiomycosis produce massive amount of propagules, i.e., zoospores. Because chytrids are small in size and lack conspicuous morphological features, a situation that makes them hardly distinguishable from many flagellated protists such as the sessile choanoflagellates or bicosoecids, their functional roles, primarily as saprotrophs or parasites, remain most of the time cryptic in classical microscopy studies (Lefèvre et al., 2007, 2008; Sime-Ngando et al., 2011). Previously, the modes of nutrition for all heterotrophic flagellates in the plankton were thought to be restricted to bacterivory, but zoosporic fungi are not bacterivores (i.e., bacterial feeders). It is now clearly evident that not all heterotrophic flagellates thriving in pelagic systems are either protists or bacterivores as previously thought, and that parasitism and saprophytism from fungal flagellates might represent important potential functions in these ecosystems. Fungal zoospores are valuable food sources for zooplankton because cytoplasm of chytrids contains storage carbohydrates such as glycogen, storage proteins, a wide range of fatty acids, phospholipids, sterols, and other lipids (Gleason et al., 2009; Sime-Ngando, in press). When chytrids reproduce, most of the cytoplasm is converted into zoospores which swim away to colonize new substrates or infect new hosts. Lipids are considered to be high energy compounds, some of which are important for energy storage. Indeed, lipids are present mainly in the form of endogenous reserves, often as membrane bound vesicles called lipoid globules which can easily be seen in the cytoplasm of fungal zoospores with both the light and electron microscopes (Gleason and Lilje, 2009; Sime-Ngando, in press). The size and numbers of lipoid globules within zoospores varied and their ultrastructure is complex. The chemical composition of lipids, including both fatty acids and sterols, has been characterized in a number of genera of zoosporic fungi. These endogenous reserves are consumed during the motile phase of the zoospores. They presumably provide energy for the movement of flagella during the motile phase which can last for up to several hours, as well as for the attachment and germination of zoospores on the appropriate substrates or hosts (Figure 2). Besides, many zoosporic fungi can grow in the laboratory on minimal synthetic media containing one carbon source such as cellulose, xylan, starch, or chitin along with salts containing nitrate, sulfate, and phosphate (Gleason et al., 2008; Sime-Ngando, in press). This establishes fungi as potential competitors of bacteria and primary producers for essential minerals (Figure 2).

There are other significant functions for the high energy compounds found in fungal zoospores, especially as food resources for zooplankton and probably for many other consumers in aquatic ecosystems. Fungal spores and hyphae in general are known to be eaten by a large number of different consumers in both aquatic and soil ecosystems, including a variety of mycophagous protozoa such as amebae and flagellates, detritivores, grazers such as filter feeding zooplankton, and benthic suspension feeders (Gleason et al., 2009; Sime-Ngando, in press). Since most of these consumers do not discriminate between food resources except by size, we would expect zoospores as well as hyphae and non-motile spores to be eaten by many of these consumers, although published records are lacking. Fungal zoospores are well within the range of a good particle size for zooplankton feeding behavior and consequently, when fed upon, transfer matter to higher trophic levels in the food chain. For example, zoospores are efficiently grazed by crustacean zooplankton such as Daphnia spp. (Kagami et al., 2007a,b, 2011; Sime-Ngando, in press), before they grow into a mature thallus (i.e., body). Thus zoospores may provide organic compounds containing nitrogen, phosphorus and sulfur, mineral ions, and vitamins to grazing zooplankton (Figure 2).

More interestingly, zoospores are a particularly good food source because of their nutritional qualities. Presumably many consumers must obtain at least some essential nutrients from their food sources because these compounds cannot be produced *de novo*. One example is found in the cladoceran *Daphnia*. Recent research has shown that zoospores of the parasitic chytrid, *Zygorhizidium*, are quite rich in polyunsaturated fatty acids (PUFAs) and cholesterols, which are essential nutrients for the growth of *Daphnia* (Kagami et al., 2007a,b). These zoospores are found to facilitate the trophic transfer from the inedible large diatom hosts, *Asterionella* sp., and the growth of *Daphnia*. PUFAs and cholesterol are known to promote growth and reproduction in crustacea. This phenomenon, known as the “trophic upgrading concept” (Sime-Ngando et al., 2011; Sime-Ngando, in press), is of significant importance in the aquatic food webs because it highlights not only the quantity but also the quality of the matter being transferred via fungal zoospores (Figure 2).

## Impact of Phytoplankton Chytridiomycosis on the Food Web Properties

Given that food webs are central to ecological concepts (Pascual and Dunne, 2006), it is important to establish the role of parasites in the structure and function of food webs. In theory, parasites can have a variety of effects. Lafferty et al. (2006, 2008) suggested that parasites affect food web properties and topology since they double connectance (defined as the number of observed links divided by the number of possible links) and quadruple the number of links. Others have postulated that parasites drive an increase in species richness, trophic levels, and trophic chain length of the food web (Huxham et al., 1995; Thompson et al., 2005). These properties may stabilize community structure (Hudson et al., 2006). However, the potential effects of parasites on food web stability is a complex and unresolved issue since the concept of stability is the center of a perhaps infinite debate in community ecology (Elton, 1958; May, 1972; Pimm, 1984; McCann, 2000; Hosack et al., 2009; O’Gorman and Emmerson, 2009). Based on the ideas of May (1973), parasites should lead to a destabilized trophic network because they increase species diversity and the connectance. In addition, adding parasites to food webs extends the length of trophic chains, which can decrease food web stability (Williams and Martinez, 2004). However, the addition of long loops of weak interactions, which may be a characteristic of parasites with complex life cycles, might offset the destabilizing effects of increased connectance (Neutel et al., 2002).

**Figure 3:**
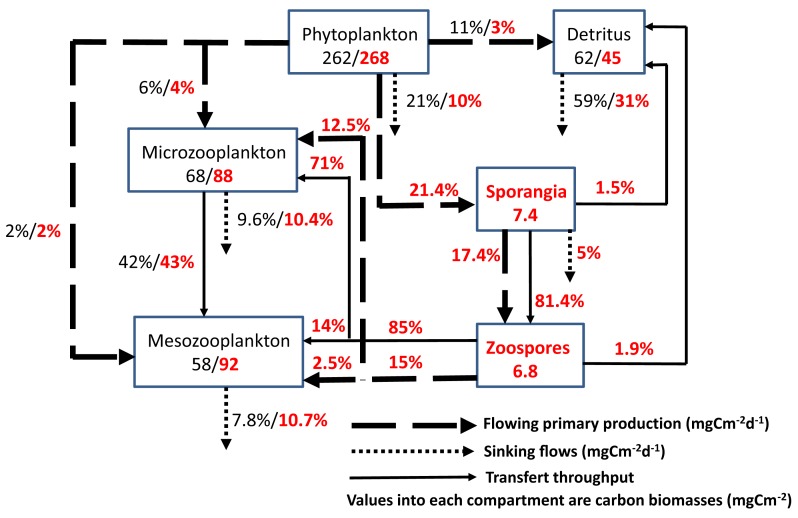
**Impact of parasitic chytrids on the microbial loop: flowing and sinking carbon from the gross primary production of phytoplankton (>20 μm) during the spring diatom bloom in the oligo-mesotrophic Lake Pavin, France**. The effects of infective fungal sporangia and their propagules (zoospores) are highlighted in red color. The diagram corresponds to steady state models of the euphotic zone of the lake generated from a linear inverse modeling analysis. For more details, see the main text. Modified from Grami et al. (2011).

To investigate ecosystem properties and ecological theories, the application of mathematical tools, such as models, is useful and allows trophic network representation through carbon flows (Figure 3). In the absence of quantification of the flows induced by fungal parasites of phytoplankton, we recently simulate their potential role in the plankton food web of the Lake Biwa, Japan (Niquil et al., 2011). The presence of this indirect pathway channeling microphytoplankton production to the consumers via the fungi leads to an enhancement of the trophic efficiency index, and a decrease of the ratio detritivory: herbivory. The results suggested that the food web relies less on the consumption of detritus, and that the transfer of carbon to higher trophic levels is higher than estimated without taking into account the parasites. Due to the lack of data quantifying carbon transfer through parasitism in pelagic ecosystems, no attempt was made to build model based on field estimated flows. Thus, the roles and ecological implications of chytrid infections of phytoplankton remain to be fully explored for aquatic microbial food webs.

As a first attempt, we recently provided such exercise for the first time, by adding parasitic chytrids of phytoplankton as an individualized compartment in a well studied pelagic food web and quantify their impact on matter flow through a trophic network (Grami et al., 2011). To describe the food web, models representative of carbon flows were built, including chytrid parasitism and the amount of primary production channeled in food web via chytrid infection. Carbon flows between the complete food web including parasitic chytrids (MWC, Model with Chytrids) were compared to the same model that did not consider the presence of chytrids and the resulting flows (MWOC, Model without Chytrid), as traditionally done in previous plankton food web analysis (e.g., Niquil et al., 2006). MWC and MWOC models were constructed on the basis of the same data set corresponding to the spring bloom period in Lake Pavin (i.e., March–June 2007). These models were built using the Linear Inverse Modeling procedure (LIM, Vézina and Platt, 1988) recently modified into the LIM-Monte Carlo Markov Chain (LIM-MCMC; Van den Meersche et al., 2009). This method allow reconstruction of missing flow values and alleviates the problem of under sampling using the principle of conservation of mass, i.e., the quantity of carbon coming into each compartment considered as equal to the amount leaving it (Vézina and Platt, 1988). Thanks to recent development of the inverse analysis into LIM – MCMC, a probability density function covering the range of possible values was generated for each flow. The results of this exercise are summarized in Figure 3 where the inclusion of the two life stages of chytrid parasites of microphytoplankton (>20 μm) increases the number of compartments and flows. These parasites were able to short-circuit about 20% of the gross primary production, of which 15% is transferred to grazers with high throughput (Figure 3).

In addition, for each calculated set of flows generated by the Linear Inverse Modeling procedure, there is a set of calculated indices which allows application of statistical tests. The flows obtained from the models were used for calculations of Ecological Network Analysis indices that characterize the structure of the food web, and help reveal emergent properties (Ulanowicz, 1986, 1997, 2003; Ulanowicz et al., 2009). The use of ecological indices moreover, allows an indirect evaluation of the effects of network properties on the stability of the ecosystem, as several authors have proposed theoretical links between structural properties and local stability (cf. Ulanowicz, 2003). On these bases, the model results support recent theories on the probable impact of parasites on food web function. In the lake, during spring, when “inedible” algae (unexploited by planktonic herbivores) were the dominant primary producers, the epidemic growth of chytrid parasites significantly reduced the sedimentation loss of algal carbon from 21 to 10% of gross primary production (Figure 3). Furthermore, from the review of some theories about the potential influence of parasites on ecological network properties, we argue that parasitism contributes to longer carbon path lengths, higher levels of activity and specialization, and lower recycling. We then conclude that considering the “structural asymmetry” hypothesis as a stabilizing pattern, chytrids should contribute to the stability of aquatic food webs (Grami et al., 2011).

## Conclusion

Chytrid symbionts and the related trophic modes, primarily parasitism, are omnipresent in aquatic ecosystems, including marine habitats (Gleason et al., 2011). In these ecosystems, they are diversified, with different taxa featured by different biological characteristics and requirements that determine their distributions in relation to environmental parameters but primarily to the seasonal dynamics of their phytoplankton hosts. The host abundance but also the host cell size and biomass likely establish the threshold limit for the critical prevalence of infection and the related decline in host communities. Related ecological implications are huge, because chytrid parasites can kill their hosts, release substrates for microbial processes, and provide nutrient-rich particles as zoospores and short fragments of filamentous hosts for the grazer food chain. This implies that cyanobacterial blooms, and other large size inedible phytoplankton blooms as well, may not totally represent trophic bottlenecks. Based on the observation that phytoplankton fungal parasitism preferentially impacts the larger size species (i.e., characteristics of climax populations), chytrid epidemics represent an important driving factor in phytoplankton successions and maturation, in addition to the sole seasonal forcing. The activity of chytrid parasites of phytoplankton thus represents an important but as yet overlooked ecological driving force in aquatic food web dynamics. In addition to being able to resist adverse conditions and use different sources of carbon and nutrients, chytrid parasites can indeed affect the plankton food web functions and ecosystem properties and topology, such as stability and trophic transfer efficiency. We are perhaps approaching a new paradigm-shift point in the development of aquatic microbial ecology (Sime-Ngando and Niquil, 2011).

However, the available study on phytoplankton chytridiomycosis remain restricted to a few temperate lakes, and extensive studies in the world’s aquatic ecosystems, at wide geographical and time scales, are needed. Besides, the identification of chytrid species based on phenotypic features requires time and experience, and chytrid diversity provided this way probably is an underestimate. In this context, the increasing development of molecular tools is promising and will, in the near future, improves the linkage of cell identity and function, which is critical for an accurate assessment that includes microbial parasites in the carbon flows, and the related biogeochemical cycling in aquatic ecosystems (Jobard et al., 2012). Furthermore, parasitic lifestyle is generally highly subtle and can, for example, control competition by dominant species for resources, thereby promoting species coexistence and diversity. Parasites can also form long-lived associations with hosts, reducing their fitness for survival, or allowing infected hosts to remain strong competitors, although few models exist for microbial fungus-host systems.

## Conflict of Interest Statement

The author declares that the research was conducted in the absence of any commercial or financial relationships that could be construed as a potential conflict of interest.
